# Loss of Histone H3 Methylation at Lysine 4 Triggers Apoptosis in *Saccharomyces cerevisiae*

**DOI:** 10.1371/journal.pgen.1004095

**Published:** 2014-01-30

**Authors:** David Walter, Anja Matter, Birthe Fahrenkrog

**Affiliations:** 1 M.E. Müller Institute for Structural Biology, Biozentrum, University of Basel, Basel, Switzerland; 2 Institute for Molecular Biology and Medicine, Université Libre de Bruxelles, Charleroi, Belgium; Buck Institute for Research on Aging, United States of America

## Abstract

Monoubiquitination of histone H2B lysine 123 regulates methylation of histone H3 lysine 4 (H3K4) and 79 (H3K79) and the lack of H2B ubiquitination in *Saccharomyces cerevisiae* coincides with metacaspase-dependent apoptosis. Here, we discovered that loss of H3K4 methylation due to depletion of the methyltransferase Set1p (or the two COMPASS subunits Spp1p and Bre2p, respectively) leads to enhanced cell death during chronological aging and increased sensitivity to apoptosis induction. In contrast, loss of H3K79 methylation due to *DOT1* disruption only slightly affects yeast survival. *SET1* depleted cells accumulate DNA damage and co-disruption of Dot1p, the DNA damage adaptor protein Rad9p, the endonuclease Nuc1p, and the metacaspase Yca1p, respectively, impedes their early death. Furthermore, aged and dying wild-type cells lose H3K4 methylation, whereas depletion of the H3K4 demethylase Jhd2p improves survival, indicating that loss of H3K4 methylation is an important trigger for cell death in *S. cerevisiae*. Given the evolutionary conservation of H3K4 methylation this likely plays a role in apoptosis regulation in a wide range of organisms.

## Author Summary

Covalent histone modifications alter chromatin structure and DNA accessibility, which is playing important roles in a wide range of DNA-based processes, such as transcription regulation and DNA repair, but also cell division and apoptosis. Apoptosis is the most common form of programmed cell death and plays important roles in the development and cellular homeostasis of all metazoans. Deregulation of apoptosis contributes to the pathogenesis of multiple diseases including autoimmune, neoplastic and neurodegenerative disorders. The budding yeast *Saccharomyces cerevisiae* has progressively evolved as model to study the mechanisms of apoptotic regulation, and we study here the role of an evolutionary conserved trans-histone crosstalk, in particular histone methylation, in apoptotic signaling in yeast. We have identified a novel trigger for cell death in yeast and due to the strong evolutionary conservation our findings may apply to human cells and may be of importance for understanding the molecular mechanism underlying a specific subtype of acute leukemia.

## Introduction

Apoptosis is the most common form of programmed cell death and plays important roles in the development and cellular homeostasis of all metazoans. Deregulation of apoptosis contributes to the pathogenesis of multiple diseases including autoimmune, neoplastic and neurodegenerative disorders [1]. The budding yeast *Saccharomyces cerevisiae* has progressively evolved as model to study the mechanisms of apoptotic regulation, as it had become evident that the extent of evolutionary conservation of the apoptotic core machinery makes it a suitable and attractive model system for apoptotic research. *S. cerevisiae* undergoes apoptosis when treated with various agents including hydrogen peroxide (H_2_O_2_), acetic acid and pheromone (reviewed in [2]). Physiological scenarios that trigger apoptosis in yeast are for example aging and failed mating, and chronological aging is in this respect the to date best-studied scenario [2], [3]. The chronological lifespan (CLS) is defined as the time a yeast cell can survive in a non-dividing, quiescence-like state [4], [5]. Genetic interventions with key yeast apoptotic regulators, such as Bir1p, Nma111p and Yca1p, have been described that influence the CLS of yeast cells and the appearance of the apoptotic features associated to it [6]–[10]. Particularly, disruption of the yeast metacaspase *YCA1* gene delays cell death and the formation of an apoptotic phenotype during chronological aging [8].

The activation of apoptosis results in characteristic biochemical and morphological features outside and inside the cell nucleus [11] with chromatin condensation paralleled by DNA fragmentation being one of the most important nuclear events in cells undergoing apoptosis [12]. The mechanism by which chromosomes reorganize during apoptosis is still poorly understood, but evidence exists that histone modifications contribute critically to the nuclear changes experienced by apoptotic cells. Histone modifications that have been linked to apoptosis are phosphorylation of the histone variant H2A.X at serine 139 (S139) that occurs during the formation of DNA double strand breaks under various conditions, including apoptosis [13]. Phosphorylation of histone H2B at S14 has been associated with chromatin condensation and DNA fragmentation [14]–[16]. This modification is reciprocal and deacetylation of H2B at lysine 15 (K15) is necessary to allow H2BS14 phosphorylation [17]. A similar mechanism appears to exist in yeast. Here deacetylation of H2BK11, which is characteristic for exponentially growing yeast [18], is necessary to allow phosphorylation of H2BS10, an apoptotic mark [19], [20]. Therefore, the cis-crosstalk between H2B acetylation and phosphorylation appears evolutionary conserved in apoptosis. Phosphorylation of H2A at serine 129 is increasing in yeast cells undergoing H_2_O_2_-induced apoptosis and it is paralleled by a decrease in H3 tyrosine 45 phosphorylation [21], pinpointing to a trans-histone crosstalk related to apoptosis in yeast.

An evolutionary conserved trans-histone crosstalk, which thus far has not been linked to apoptosis, is the regulation of H3K4 and H3K79 methylation by H2BK123 ubiquitination [22]. This trans-histone crosstalk has gathered much attention in recent years, since H3K4 and H3K79 methylation have been implicated in many nuclear processes, such as transcription activation and repression, DNA replication, recombination and repair [22], [23]. The Set1p-containing complex COMPASS acts as H3K4 methyltransferase, and this methyl mark is important for transcriptional activation [24]–[27] as well as silencing at telomeres [27], [28] and rDNA loci [29]–[31]. Methylation of H3K79 is mediated by the histone methyltransferase Dot1p and is essential for efficient silencing near telomeres, rDNA loci, and the yeast mating type loci [28]. Moreover, H3K79 methylation is critical for proper DNA damage response (DDR) [32], [33], as it is prerequisite for Rad9p (53BP1) recruitment [34]. H2B ubiquitination, which is dependent on the ubiquitin conjugase Rad6p and the E3 ligase Bre1p [35]–[37], has been implicated in DNA repair and DDR [33], [38] and we have previously shown that lack of H2B ubiquitination causes metacaspase-dependent apoptosis in *S. cerevisiae* [39]. H2B ubiquitination is furthermore known to render chromatin resistant to nuclease digestion and its absence is consequently causing increased nuclease sensitivity [22], in keeping with the observed increase in apoptosis.

In this study we analyzed whether apoptosis sensitivity of cells that lack H2B ubiquitination is dependent on a lack of H3K4 and/or H3K79 methylation. We show that *Δset1* cells are susceptible to Yca1p-dependent apoptosis, whereas *DOT1* disruption affects apoptosis to a lesser extent. We moreover found that Dot1p along with the checkpoint kinase Rad9p is critical for cell death of *Δset1* cells. Apoptosis sensitivity of *Δset1* cells can be rescued by deleting the yeast homolog of endonuclease G, Nuc1p, suggesting that loss of H3K4 methylation in the presence of H3K79 methylation and the kinase Rad9p enhances chromatin accessibility to endonuclease digestion. Wild-type, but not *dot1Δ* cells, lose H3K4 methylation during chronological aging coinciding with a shorter lifespan, indicating that the loss of H3K4 methylation is an important trigger for apoptotic cell death.

## Results

### *SET1* disruption causes apoptosis in a partly Yca1p-dependent manner

**Figure 1:**
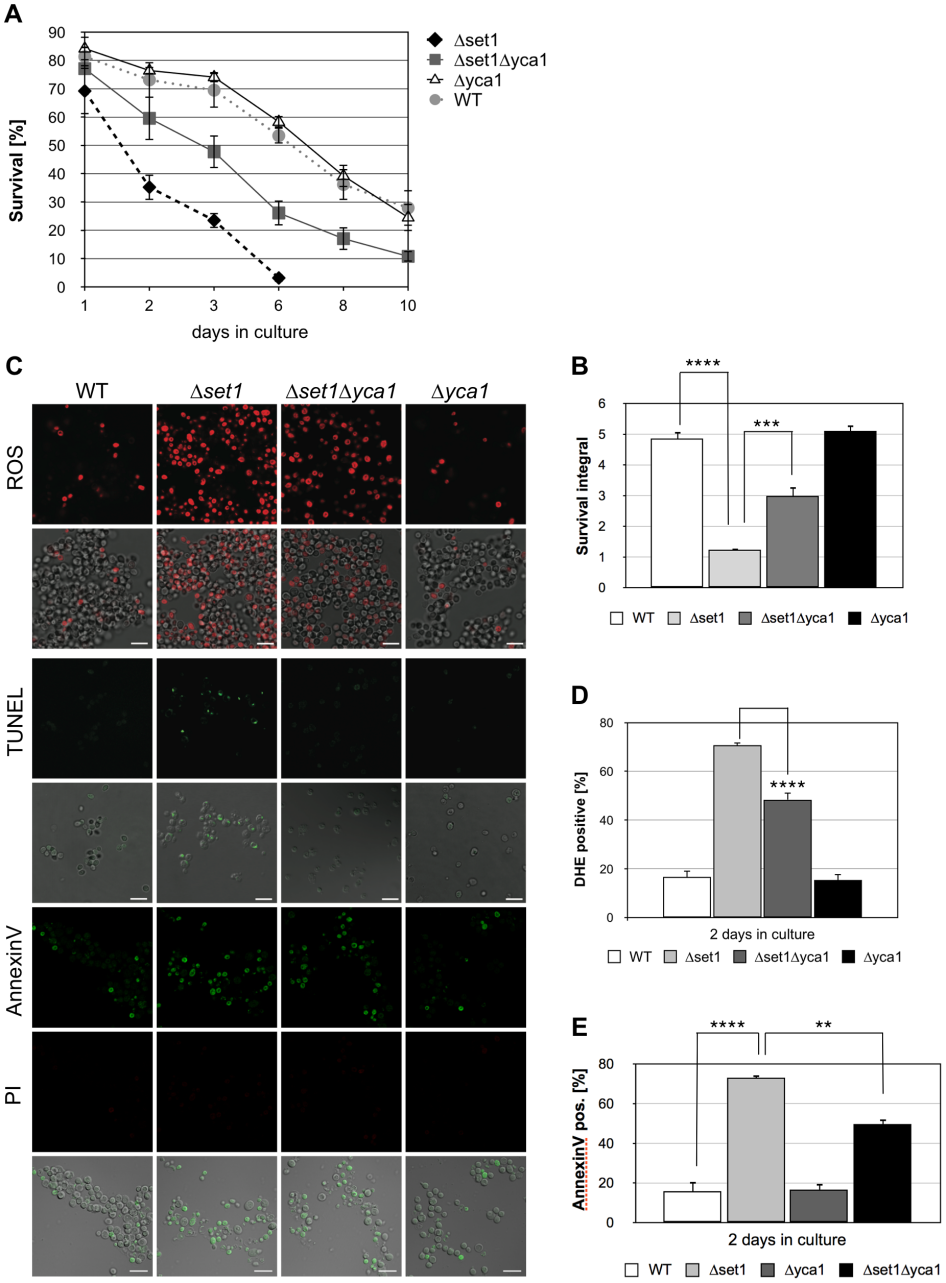
*SET1* disruption causes Yca1p-dependent cell death during chronological aging. (**A**) Survival of wild-type (WT), *Δyca1*, *Δset1* and *Δset1Δyca1* cells was determined by clonogenicity during chronological aging. Data represent mean ± SD (*n* = 3). (**B**) Integrals under the life span curves were determined (see Materials and methods). Data represent mean ± SD (*n* = 3, ****P<0.0001, ***P<0.001). (**C**) ROS accumulation, DNA fragmentation and phosphatidylserine exposure on the cell membrane in WT, *Δyca1*, *Δset1* and *Δset1Δyca1* cells after 2 days in culture determined by DHE, TUNEL and Annexin V staining, respectively. Propidium iodide (PI) staining was utilized to discriminate between apoptotic (PI negative) and necrotic (PI positive) cell death. Scale bars, 10 µm. (**D**) DHE-positive cells were quantified after 2 days in culture using flow cytometry. In each experiment, 10.000 cells were evaluated. Data represent mean ± SD (*n* = 3, ****P<0.0001). (**E**) Annexin V-positive cells were quantified after 2 days in culture by fluorescence microscopy. In each experiment, 1000–2000 cells were evaluated. Data represent mean ± SD, ****P<0.0001, **P<0.01.

Histone H3K4 methylation is mediated by the methyltransferase Set1p [27]. To test whether a lack of H3K4 methylation predisposes yeast to apoptotic stimuli, we analyzed the apoptosis sensitivity of *Δset1* cells. Chronological aging is to date the best-studied physiological scenario of apoptosis induction in yeast and we therefore studied the effect of *SET1* disruption on the chronological lifespan of yeast cells (see Material and Methods). We found that *Δset1* cells showed an early onset of cell death during chronological aging when compared to wild-type cells (Figure 1). Almost 100% of *Δset1* cells were dead after about 6 days in culture, whereas ∼30% of the wild-type cells were surviving for more than 10 days (Figure 1A). To quantify the difference in life span, we calculated the integral of the survival curve for wild-type and *Δset1* cells, which allows to determine the survival differences for the two strains over the time course of the experiment [40]. The survival integral of *Δ* *set1* cells (integral 1.2) is significantly smaller than the integral for wild-type cells (integral 4.9) (Figure 1B). Next, we asked whether the death of *SET1* disrupted cells is of apoptotic nature. Apoptosis (but also necrosis) is frequently accompanied by an accumulation of reactive oxygen species (ROS), which is an early step in the apoptotic process [41]. Staining with dihydroethidium (DHE) was used to visualize accumulation of ROS. DNA fragmentation was detected by using TUNEL staining, and combined Annexin V/propidium iodide (PI) staining was used to detect the cell surface exposure of phosphatidylserine, an early apoptotic event. PI staining further allows the discrimination between apoptotic (PI negative) and necrotic (PI positive) cell death. ROS accumulation was determined after 2 days in culture, when *Δset1* cells showed survival of about 35% compared to ∼75% of wild-type cells (Figure 1A), as determined by clonogenicity. At this time point about 70% of *Δset1* cells were DHE positive, but only ∼20% of wild-type cells (Figure 1C and D; Table 1). Consistently, *Δset1* cells unlike wild-type cells show apoptotic DNA fragmentation, as determined by TUNEL staining (Figure 1C). Moreover, about 73% of *Δset1* cells were stained positive for Annexin V, but negative for PI, compared to about 16% of wild-type cells (Figure 1C and E). Together our data demonstrate that cells lacking *set1* have a reduced CLS and their death is predominantly of apoptotic nature.

**Table 1:** Survival integrals and ROS accumulation for all assays in this study.

	Strain	Survival integral	DHE positive [%]
**Figure 1**	WT	4.9±0.2	16.7±2.5
	*Δset1*	1.2±0.1	70.7±1.2
	*Δset1Δyca1*	2.9±0.3	48.3±2.9
	*Δyca1*	5.1±0.2	15.3±2.5
**Figure 2**	WT	4.9±0.2	45.0±1.0
	*Δdot1*	5.8±0.2	34.0±1.0
	*Δdot1Δyca1*	5.1±0.1	38.7±1.5
	*Δyca1*	5.1±0.2	36.3±1.5
**Figure 3**	WT	5.3±0.2	16.6±2.5
	*Δset1*	2.6±0.4	70.6±1.1
	*Δset1Δdot1*	4.6±0.7	47.0±5.3
	*Δset1Δdot1Δyca1*	4.9±0.4	60.3±3.0
**Figure 4**	WT	3.3±0.2	16.7±2.5
	*Δset1*	1.2±0.1	70.7±1.2
	*Δset1Δrad9*	2.4±0.1	45.0±2.6
	*Δrad9*	3.3±0.1	11.5±1.4
	*Δset1Δdot1*	2.7±0.1	47.0±5.3
	*Δset1Δdot1Δrad9*	2.3±0.2	46.0±2.0
**Figure 5**	WT	6.0±0.2	24.9±3.8
	*Δset1*	3.8±0.4	54.1±8.9
	*Δnuc1*	5.1±0.5	45.2±7.4
	*Δset1Δnuc1*	6.6±1.0	23.8±7.9
**Figure 6**	WT	6.0±0.5	48.5±5.6
	*Δset1*	2.8±0.2	71.3±9.6
	*Δjhd2*	7.3±0.8	43.6±9.4
**Figure 7**	WT	7.3±0.5	10.3±3.5
	*Δset1*	3.5±0.1	30.0±3.4
	*Δspp1*	3.5±0.1	22.7±1.5
	*Δbre2*	4.3±0.7	21.3±1.9
	H3 WT	2.5±0.2	45.2±6.2
	H3 K4A	1.6±0.2	64.7±7.6
	H3 K79A	3.1±0.1	49.3±5.9
**Figure 8**	WT		3.2±2.6
	*Δset1*		22.8±1.2
	*Δyca1*		2.4±3.6
	*Δset1Δyca1*		11.7±4.3

Listed are mean ± SD. ND, not determined.

Apoptosis in yeast can occur in a metacaspase-dependent or metacaspase-independent manner [42]. We thus asked whether the metacaspase Yca1p is required for the death of *Δset1* cells. Therefore, *Δset1Δyca1* double disruptants were generated and their survival was monitored during chronological aging. As shown in Figure 1A and B, deletion of Y*CA1* in the *SET1*-deleted background resulted in significantly better survival (integral 3.0) when compared to *Δset1* cells (integral 1.2). The improved survival of the double mutant is accompanied by a significant reduction of ROS accumulation and phosphatidylserine exposure on the cell membrane (Figure 1C–E). Moreover, unlike *Δset1* cells, but similar to *Δyca1* cells, *Δset1Δyca1* cells did not exhibit apoptotic DNA fragmentation as detected by TUNEL labeling (Figure 1C). Thus, the apoptotic death of *Δset1* cells is in part Yca1p-dependent.

### *DOT1* is required for Yca1p-dependent apoptosis

**Figure 2:**
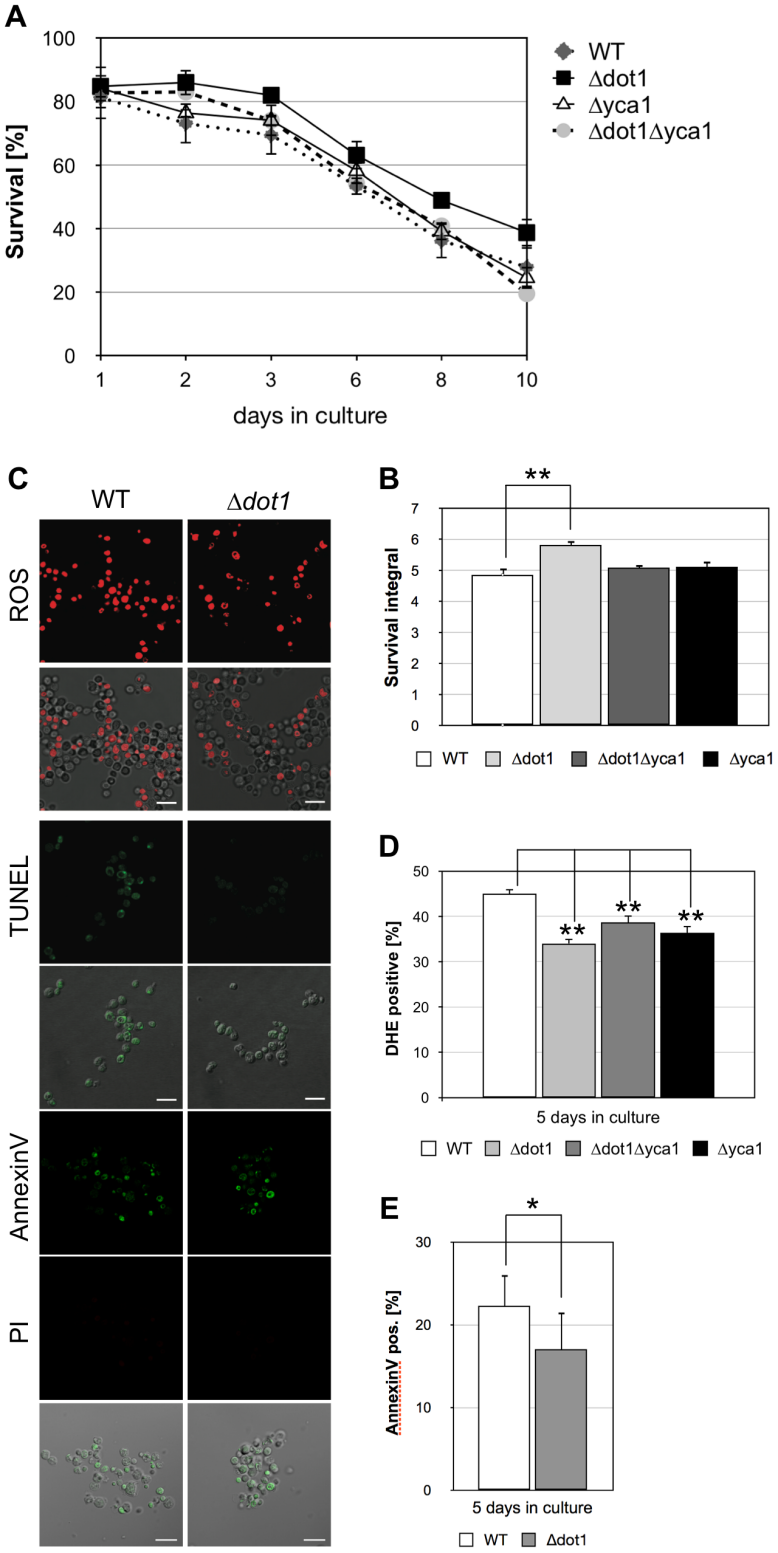
*DOT1* disruption positively influences Yca1p-dependent cell death. (**A**) Survival of WT, *Δdot1, Δyca1* and *Δdot1Δyca1* cells was determined by clonogenicity during chronological aging. Data represent mean ± SD (*n* = 3). (**B**) Integrals under the life span curves were determined. Data represent mean ± SD (*n* = 3, **P<0.01). (**C**) ROS accumulation, DNA fragmentation, and phosphatidylserine exposure on the cell membrane in WT and *Δdot1* cells after 5 days in culture determined by DHE, TUNEL and Annexin V/PI staining, respectively. Scale bars, 10 µm. (**D**) DHE-positive WT, *Δdot1*, *Δyca1* and *Δdot1Δyca1* cells were quantified after 5 days in culture using flow cytometry. In each experiment, 10.000 cells were evaluated. Data represent mean ± SD (*n* = 3, **P<0.01). (**E**) Annexin V-positive cells were quantified after 5 days in culture by fluorescence microscopy. In each experiment, 1000–2000 cells were evaluated. The difference between wild-type and *Δdot1* cells are statistically significant (*P<0.05).

Histone H2B ubiquitination is not only a prerequisite for H3K4 methylation but also for H3K79 methylation. We next asked whether the lack of Dot1p and H3K79 methylation also influences apoptotic cell death of *S. cerevisiae*. *DOT1* disrupted cells showed better survival during chronological aging when compared to wild-type cells (Figure 2A and B). The effect of *dot1* disruption on cell survival is modest, but statistically relevant with survival integrals of 5.8 for *Δdot1* cells versus 4.9 for the wild-type (Figure 2B). Consistently, the *Δdot1* strain exhibited less ROS accumulation than wild-type cells, less apoptotic DNA fragmentation as detected by TUNEL labeling, and a slight decrease in the exposure of phosphatidylserine on the cell membrane (Figure 2C–E; Table 1). To further underline the statistical relevance of the survival advantage of *Δdot1* cells as compared to wild-type cells, we normalized the survival of both strains to survival at day 2 to ensure that all yeast cells have reached stationary phase. Again, we found that the difference in cell survival between wild-type and *dot1Δ* cells is statistically relevant (Figure S2A and B). Together our data suggest that Dot1p in opposite to Set1p may protect against cell death.

Next, we asked whether Dot1p promoted cell death depends on Yca1p and generated a *Δdot1Δyca1* double mutant to analyze its viability. A better survival of the double mutant as compared to the single mutant cells is expected, if Dot1p and Yca1p act independently. However, *Δdot1Δyca1*, *Δdot1* and *Δyca1* cells exhibited similar viability during chronological aging (Figure 2A and B) with similar survival integrals as wild-type cells (Figure 2B) and similar ROS accumulation (Figure 2D; Table 1). Together these data suggest that Dot1p and Yca1p act within the same apoptotic pathway as pro-apoptotic proteins.

### Dot1p is required for apoptosis in *Δset1* cells

**Figure 3:**
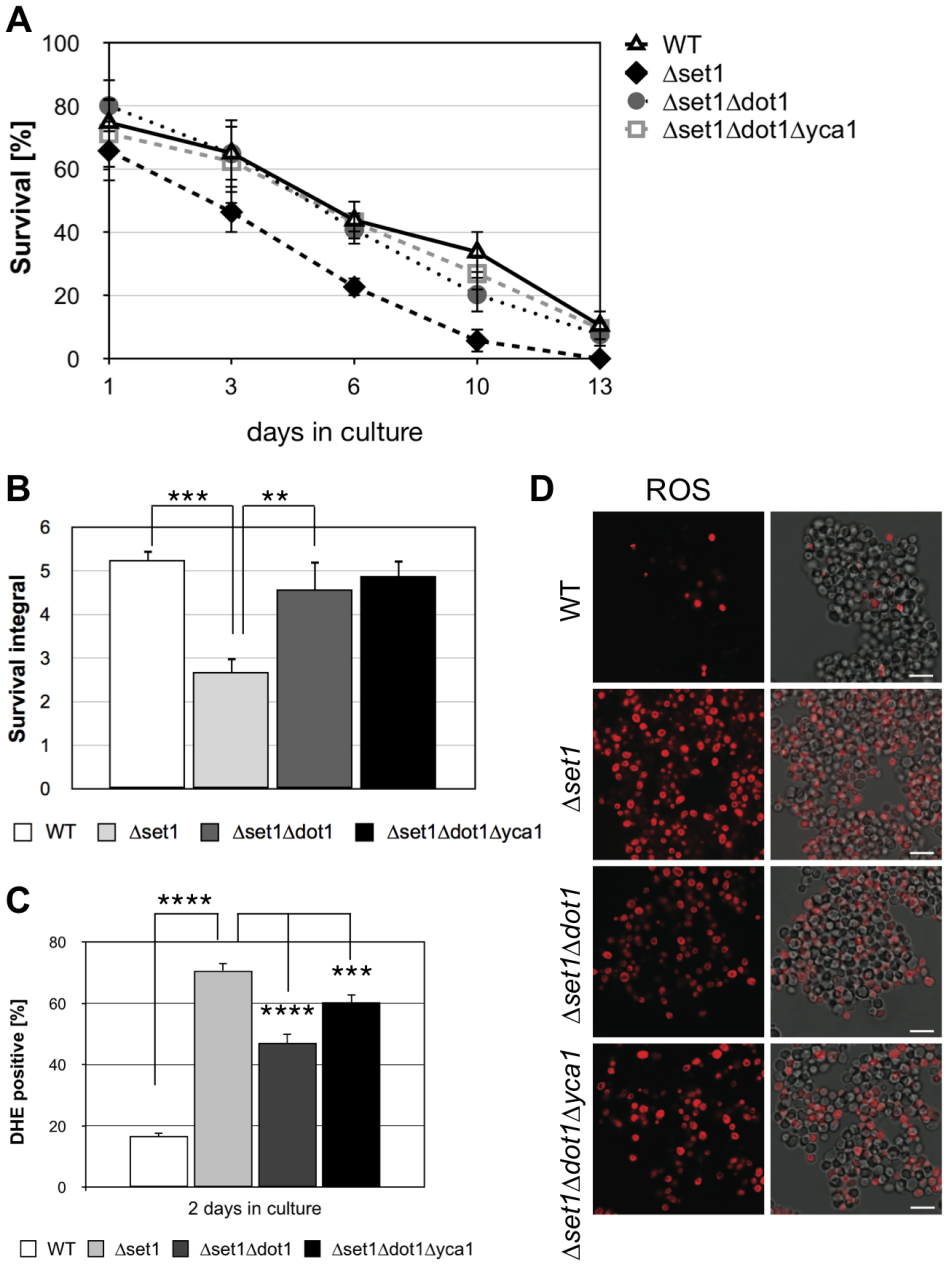
Dot1p is required for Yca1p-dependent cell death of *Δset1* cells. (**A**) Survival of WT, *Δyca1*, *Δdot1*, *Δset1*, *Δset1Δdot1* and *Δset1Δdot1Δyca1* cells was determined by clonogenicity during chronological aging. Data represent mean ± SD (*n* = 3). (**B**) Integrals under the life span curves were determined. Data represent mean ± SD (*n* = 3, **P<0.01, ***P<0.001). (**C**) DHE-positive cells were quantified after 2 days in culture using flow cytometry. In each experiment, 10.000 cells were evaluated. Data represent mean ± SD (*n* = 4, ***P<0.001, ****P<0.0001). (**D**) ROS accumulation in WT, *Δyca1*, *Δdot1*, *Δset1*, *Δset1Δdot1* and *Δset1Δdot1Δyca1* cells after two days in culture was determined by DHE staining. Scale bars, 10 µm.

The above experiments show that Dot1p-mediated H3K79 methylation supports cell death, while Set1p-mediated H3K4 methylation confers apoptosis resistance. Next, we asked if the death of cells lacking H3K4 methylation is dependent on H3K79 methylation and generated a *Δset1Δdot1* double mutant, lacking histone H3K4 and K79 methylation. As shown in Figure 3A and B, we observed a significantly improved survival during chronological aging for the *Δset1Δdot1* double mutant (integral 4.6) as compared to *Δset1* cells (integral 2.7), with consistent ROS accumulation of only 47% for Δ*set1*Δ*dot1* cells compared to 70% of Δ*set1* cells (Figure 3C and D; Table 1). These data confirm the pro-death role of Dot1p and suggest that H3K79 methylation is important for cell death of *Δset1* cells.

Next, we asked whether or not Dot1p is required for Yca1p-dependent cell death of *Δset1* cells. We therefore generated a triple mutant *Δset1Δdot1Δyca1* strain and analyzed its survival during chronological aging. If Dot1p acts in an Yca1p-independent manner, a better survival of the triple mutant as compared to *Δset1Δdot1* cells is expected. This, however, was not the case. The triple mutant *Δset1Δdot1Δyca1* showed no better survival as compared to *Δset1Δdot1* cells and a similar number of DHE-positive cells (Figure 3A–D; Table 1). Thus, Dot1p and Yca1p act together in *Δset1* provoked cell death.

### The DNA damage adaptor protein Rad9p is required for apoptosis of *SET1*-depleted cells

**Figure 4:**
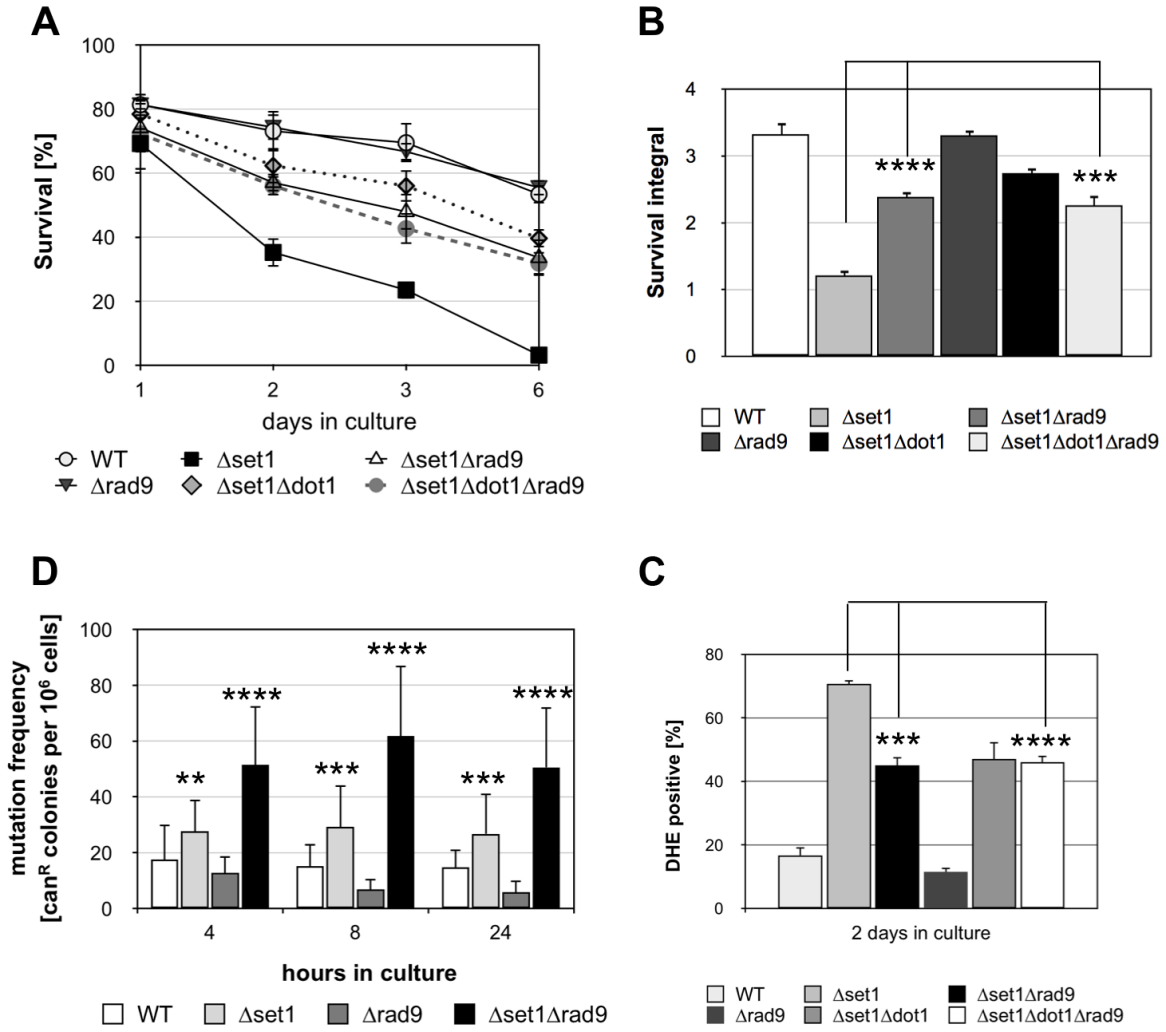
Disruption of *SET1* accelerates genome instability and Rad9p is required for apoptosis of **Δ*set1* cells. (**A**) Survival of WT, *Δset1*, *Δrad9*, *Δset1Δrad9*, *Δset1Δdot1* and *Δset1Δrad9Δdot1* cells was determined by clonogenicity during chronological aging. Data represent mean ± SD (*n* = 3). (**B**) Integrals under the life span curves were determined. Data represent mean ± SD (*n* = 3, ***P<0.001). (**C**) DHE-positive cells were quantified after 2 days in culture using flow cytometry. In each experiment, 10.000 cells were evaluated. Data represent mean ± SD (*n* = 3, ***P<0.001, ****P<0.0001). (**D**) Mutation frequency measured by resistance to canavanine in WT, *Δset1*, *Δrad9*, *Δset1Δrad9* cells. Error bars depict the standard deviation for measurements made in 5–6 independent experiments carried out in triplicates. **P<0.01, ***P<0.001, ****P<0.0001.

Dot1p and H3K79 methylation has been shown to confer yeast cells with resistance to DNA damaging agents and the loss of such histone modification causes defective DDR by impairing the function of Rad9p [32], [33]. Rad9p is an adaptor protein required for Rad53p activation [43], [44]. Interestingly, deletion of the *RAD9* gene can partially suppress lethal effects of the apoptotic *orc2*-*1* mutation in the origin recognition complex [45], suggesting that Rad9p-dependent checkpoint function is required for apoptosis induction in *orc2-1* cells. Given that Dot1p is required for Rad9p-dependent checkpoint activation, *Δdot1* cells might fail to activate apoptosis as a result of a defective checkpoint function. To test this hypothesis, we analyzed the survival of *Δset1Δrad9* cells during chronological aging and found that the disruption of *RAD9* in *Δset1* cells significantly improved viability (Figure 4A and B). Consistently, DHE-detectable ROS accumulation was reduced in *Δset1Δrad9* cells compared to *Δset1* cells (Figure 4C; Table 1). Deletion of *RAD9* in a wild-type background does not affect the survival of yeast cells and ROS production, respectively (Figure 4A–C). To test, if *set1* depleted cells in fact accumulate DNA damage in a Rad9p-dependent manner, we assayed genome stability of these cells by measuring the mutation frequency in the *CAN1* gene. Mutations in the *CAN1* gene can be monitored by increased resistance of yeast cells to the toxic amino-acid analogue canavanine and has previously been linked to shorter CLS [46]. We found that *SET1* deletion rapidly induced an increase in mutation frequency, which was further increased by a combined deletion of *SET1* and *RAD9* (Figure 4D), and maintained, but not further increased over time (Figure 4D). Interestingly, *RAD9* deletion alone does not coincide with an increased mutation frequency. Together these data indicate that Dot1p is required for apoptosis of *Δset1* cells in a Rad9p-dependent manner.

To confirm that Dot1p and Rad9p in fact act in the same apoptotic pathway, we tested the apoptosis sensitivity of a *Δset1Δrad9Δdot1* triple mutant. Compared to the *Δset1Δdot1* and *Δset1Δrad9* double mutants, respectively, a decrease in cell death for the triple mutant is expected in the case *DOT1* and *RAD9* disruption confer apoptosis resistance independent of each other. The *Δset1Δrad9Δdot1* triple mutant, however, exhibited similar survival curves, integrals and ROS accumulation as *Δset1Δdot1* and *Δset1Δrad9* cells (Figure 4A–C; Table 1). Therefore Dot1p and Rad9p act as pro-apoptotic proteins within the same pathway and the DDR machinery appears to be required for the activation of cell death of aged *Δset1* cells.

### Disruption of *NUC1* protects *Δset1* cells from cell death

**Figure 5:**
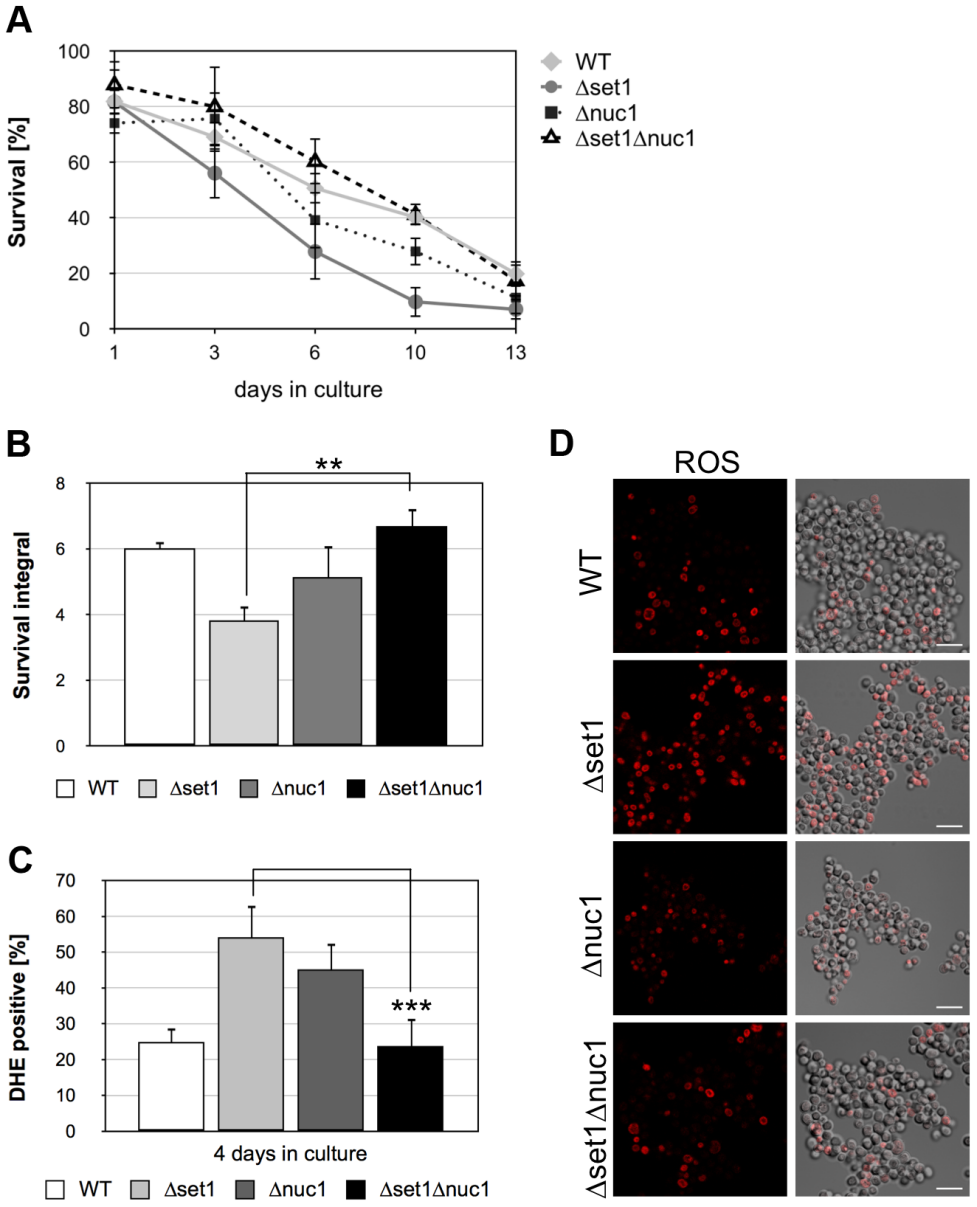
*NUC1* disruption hampers apoptosis onset and improves viability of yeast cells lacking Set1p. (**A**) Survival of WT, *Δset1*, *Δnuc1* and *Δset1Δnuc1* cells was determined by clonogenicity during chronological aging. Data represent mean ± SD (*n* = 3). (**B**) Integrals under the life span curves were determined. Data represent mean ± SD (*n* = 3, **P<0.01). (**C**) DHE-positive WT, *Δset1, Δnuc1* and *Δset1Δnuc1* cells were quantified after 4 days in culture by fluorescence microscopy. In each experiment, 800–1500 cells were evaluated. Data represent mean ± SD, ***P<0.001. (**D**) ROS accumulation in WT, *Δyca1*, *Δdot1*, *Δset1*, *Δset1Δdot1* and *Δset1Δdot1Δyca1* cells after two days in culture was determined by DHE staining and visualized by fluorescence microscopy. Scale bars, 10 µm.

Nuc1p (EndoG) is a mitochondrial nuclease that translocates into the nucleus upon apoptosis induction coinciding with DNA fragmentation [47]. As loss of H2B ubiquitination is accompanied by an increased sensitivity to nuclease digestion [22], we next asked whether the reduced viability and accelerated apoptosis of cells lacking H3K4 methylation is dependent on nuclease activity. As shown in Figure 5, deletion of *NUC1* in *Δset1* cells significantly enhanced survival and consequently the survival integrals (Figure 5 A and B; Table 1). Furthermore, deletion of *NUC1* in *Δset1* cells diminished ROS production during aging (Figure 5C and D; Table 1). Consistent with previously published data [47], aged *Δnuc1* cells showed increased cell death compared to wild-type (Figure 5A and B), which likely is of non-apoptotic nature [47], but accompanied by increased ROS production (Figure 5C). In contrast to *nuc1* disruption, deletion of the apoptosis-inducing factor *AIF1*, which also exhibits nuclease activity [7], does not rescue the apoptotic phenotype of *Δset1* cells (Figure S1).

### Loss of H3K4 methylation is the cause for apoptotic cell death

**Figure 6:**
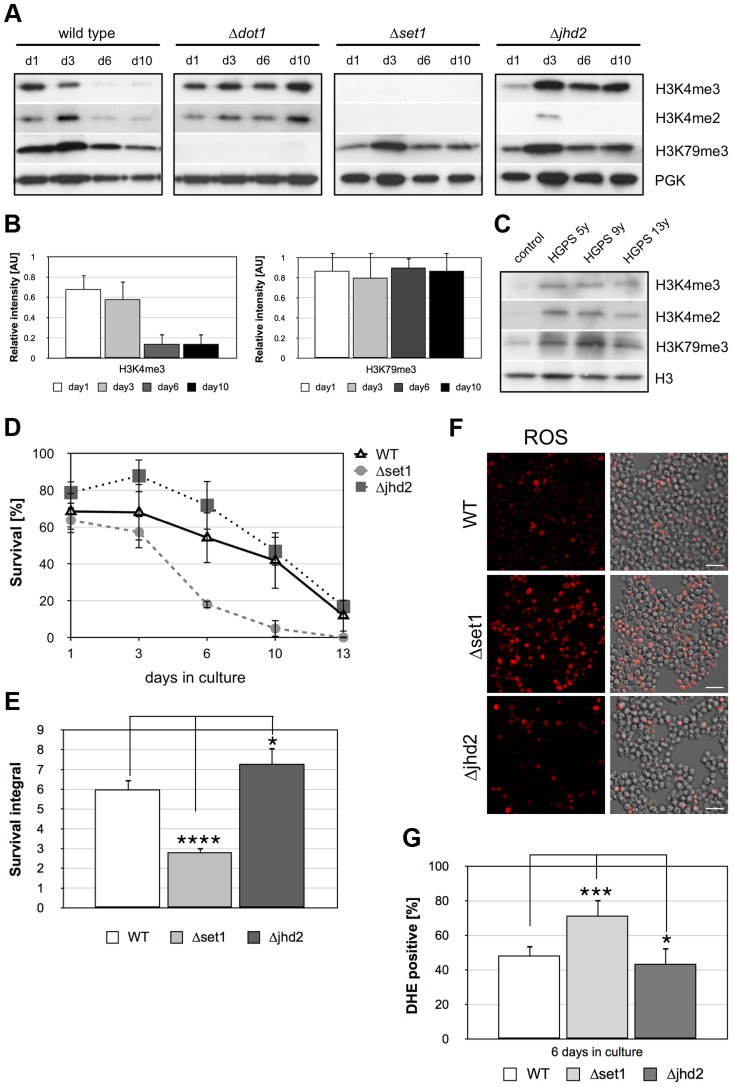
Apoptosis is associated with Dot1p-dependent loss of H3K4 methylation and preventing demethylation delays age-dependent cell death. (**A**) Western analysis of wild-type, *Δ* *dot1*, *Δset1*, and *Δjhd2* cells to monitor H3K4 and H3K79 methylation during chronological aging. Phosphoglycerolkinase (PGK) antibodies were employed as loading control. (**B**) Relative intensity of H3K4me3 and H3K79me3 levels in wild-type cells during chronological aging as determined by densitometry from three independent experiments using Image J software. (**C**) Immunoblot analysis of H3K4 and H3K79 methylation levels in lymphocytes derived from Hutchinson-Gilford progeria syndrome (HGPS) patients at the age of 5, 9 and 13 years, respectively as well as unaffected control donors. (**D**) Survival of wt, *Δset1*, and *Δjhd2* cells was determined by clonogenicity during chronological aging. Data represent mean ± SD (*n* = 3). (**E**) Integrals under the life span curves were determined. Data represent mean ± SD (*n* = 3, *P<0.05, ***P<0.001). (**F**) DHE-positive WT, *Δset1*, and *Δjhd2* cells were visualized and (**G**) quantified after 6 days in culture by fluorescence microscopy. In each experiment, 800–1300 cells were evaluated. Data represent mean ± SD, ****P<0.0001, *P<0.05. Scale bars, 10 µm.

The reduced viability of *Δ* *set1* cells during chronological aging suggests that loss of H3K4 methylation accompanies cell death of aged wild-type yeast cells. To test this possibility, we carried out Western analysis to monitor H3K4 methylation in wild-type and *Δ* *dot1* cells during chronological aging (Figure 6A). A loss of Set1p-mediated H3K4 tri- and dimethylation was observed in aging wild-type cells after 6 days in culture, but not in *Δ* *dot1* cells. Dot1p-mediated H3K79 trimethylation remained unaltered in aged wild-type cells (Figure 6A). Quantification of the H3K4me3 and the H3K79me3 levels in wild-type cells normalized to phosphoglycerolkinase (PGK) revealed an about 5-fold reduction of H3K4me3 from day 1 to day 6 and 10, while H3K79me3 levels remain equal (Figure 6B). Similar to aged *Δ* *dot1* cells, H3K4 and H3K79 methylation remained unaffected in lymphocytes derived from Hutchison-Gilford progeria syndrome (HGPS) patients (Figure 6C). HGPS is a human premature aging disease, predominantly due to mutations in the gene encoding the intermediate filament protein lamin A, and these cells are thought to be in a senescence-like state [48]. Compared to unaffected control cells, cells derived from differently aged HGPS patients (5 years old, 9 years and 13 years) show a similar increase in H3K4me3 and H3K4me2 levels. These data indicate that loss of H3K4 methylation is not a general aging-related event, but rather specific for aged yeast cells undergoing apoptosis.

If loss of H3K4 methylation in fact acts as trigger for apoptosis, one would predict that abolishing demethylation of H3K4 protects aging yeast cells from cell death. Demethylation of H3K4 is mediated by the trimethyl demethylase Jhd2p [49], [50]. To test our hypothesis, we analyzed the viability of *JHD2* deletion cells during chronological aging and found that *Δjhd2* cells showed a slightly better survival as compared to wild-type cells with larger survival integrals and reduced ROS production (Figure 6D–G; Table 1). Moreover, the differences in survival of wild-type and *Δjhd2* cells are statistically relevant when survival is normalized to day 2, when all cells have reached the postmitotic stage (Figure S2C and D). A double disruption of *JHD2* and *DOT1* has no additive effect on improvement of cell survival (data not shown), indicating that Jhd2p and Dot1p act in the same pathway. Consistent with the hypothesis that increased or stable H3K4me3 levels are advantageous for survival, H3K4me3 levels increase in aged *Δjhd2* cells from day 1 to day 3 and remain stable until day 10 (Figure 6A). We observed, however, no or undetectable H3K4me2 in *jhd2* deleted cells as in *Δset1* cells. Similar to wild-type and *Δset1* cells, H3K79me3 levels remain unaltered during aging of *Δjhd2* cells (Figure 6A). Together, our data strongly support the notion that loss of H3K4 methylation, in particular reduced trimethylation, is the cause for apoptotic death of yeast cells.

### Loss of H3K4 methylation directly sensitizes yeast cells for apoptosis

**Figure 7:**
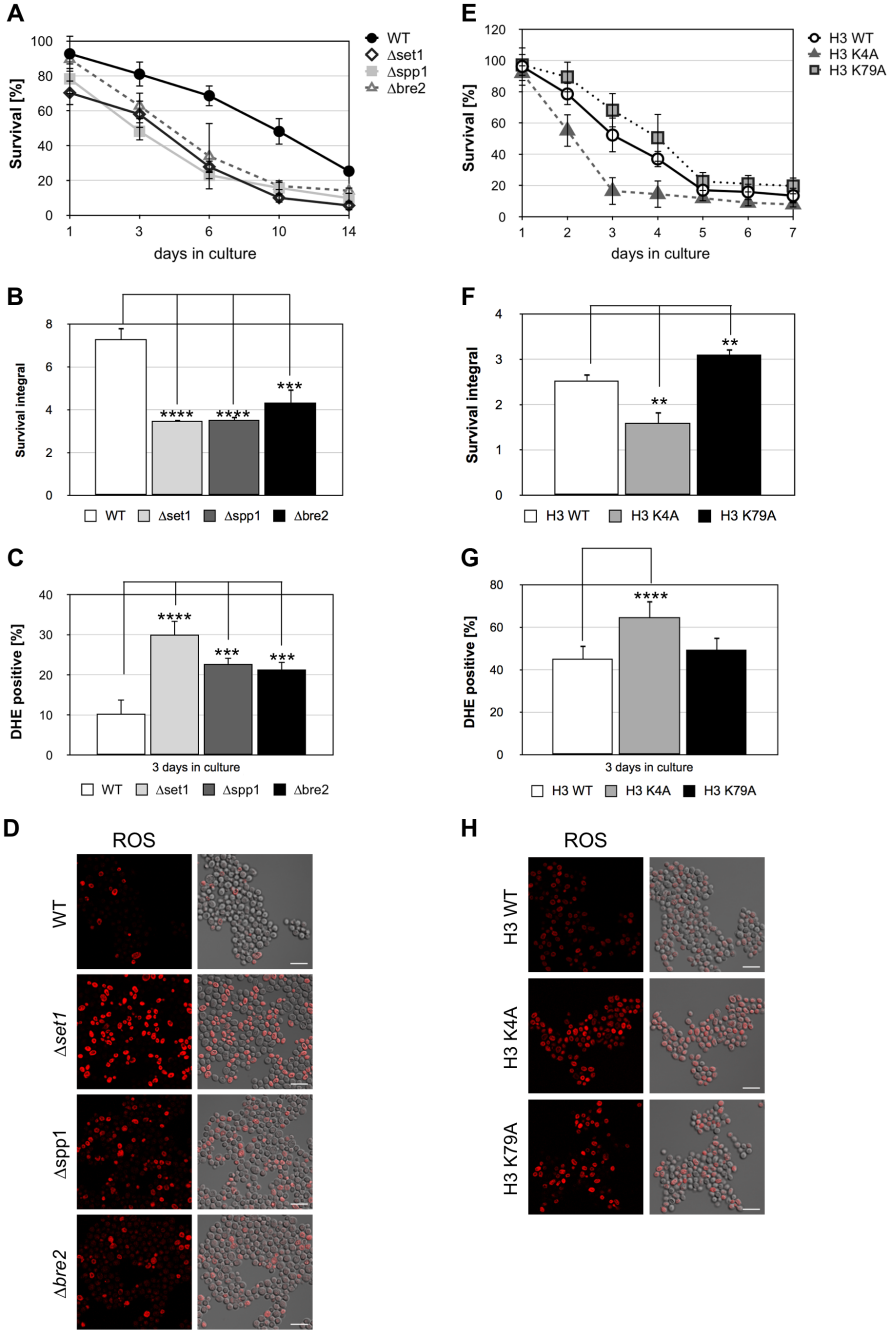
Disruption of H3K4 trimethylation enhances cell death. (**A**) Survival of wild-type (WT), *Δset1, Δspp1*, and *Δbre2* cells was determined by clonogenicity during chronological aging. Data represent mean ± SD (*n* = 3). (**B**) Integrals under the life span curves were determined. Data represent mean ± SD (*n* = 3, ***P<0.01, ****P<0.0001). (**C**) DHE-positive cells were visualized and (**D**) quantified after 3 days in culture by fluorescence microscopy. In each experiment, 5000–7000 cells were evaluated. Data represent mean ± SD, ****P<0.0001, ***P<0.001. Scale bars, 10 µm. (**E**) Survival of yeast cells expressing wild-type H3 (H3 WT), as well as cells expressing point mutations in K4 (H3 K4A) and K79 (H3 K79A) was determined by clonogenicity during chronological aging. Data represent mean ± SD (*n* = 3). (**F**) Integrals under the life span curves were determined. Data represent mean ± SD (*n* = 3, **P<0.01). (**G**) DHE-positive cells were visualized and (**H**) quantified after 3 days in culture by fluorescence microscopy. In each experiment, 2000–5000 cells were evaluated. Data represent mean ± SD, *P<0.05, ****P<0.0001. Scale bars, 10 µm.

To further strengthen the notion that loss of H3K4 trimethylation is causing the reduced viability of *Δ* *set1* cells during chronological aging, we next analyzed the viability of yeast cells lacking the two COMPASS subunits Spp1p and Bre2p, respectively. Both, Spp1p and Bre2p were previously shown to be required for proper H3K4 trimethylation [51]. Deletion of either *SPP1* or *BRE2* led to an early onset of cell death during chronological aging, similar to *SET1* deleted cells, (Figure 7A and B; Table 1) and to a boosted production of ROS (Figure 7C and D; Table 1). These data strongly support the notion that loss of H3K4 trimethylation is correlated with an early onset of apoptosis. To furthermore reveal that in fact the lack of H3K4 trimethylation is accounting for the increase in apoptotic cell death, we next tested the consequence of a point mutation in H3K4, which prevents methylation of H3 at this site, on apoptosis. To do so, we analyzed the viability of the yeast strain H3K4A, which expressses a histone H3 variant containing a lysine-to-alanine substitution at lysine 4 [52], during chronological aging. We found that these cells showed an early onset of cell death (Figure 7 E and F), similar to *Δset1*, *Δspp1*, and *Δbre2* cells. This increase in cell death of H3K4A cells coincided with enhanced ROS production (Figure 7 G and H). In contrast to H3K4A cells, H3K79A cells that lack methylation at lysine 79 showed an improved survival as compared to wild-type cells (Figure 7 E and F; Table 1). As for *Δdot1* cells (Figure 2), the effect of the K79A substitution was remote, but statistically relevant (Figure 7F). ROS levels were similar to wild-type cells, albeit a bit increased (Figure 7 F and G). Thus, loss of H3K4 trimethylation directly triggers apoptotic cell death during chronological aging, whereas loss of H3K79 trimethylation moderately improves cell survival.

**Figure 8:**
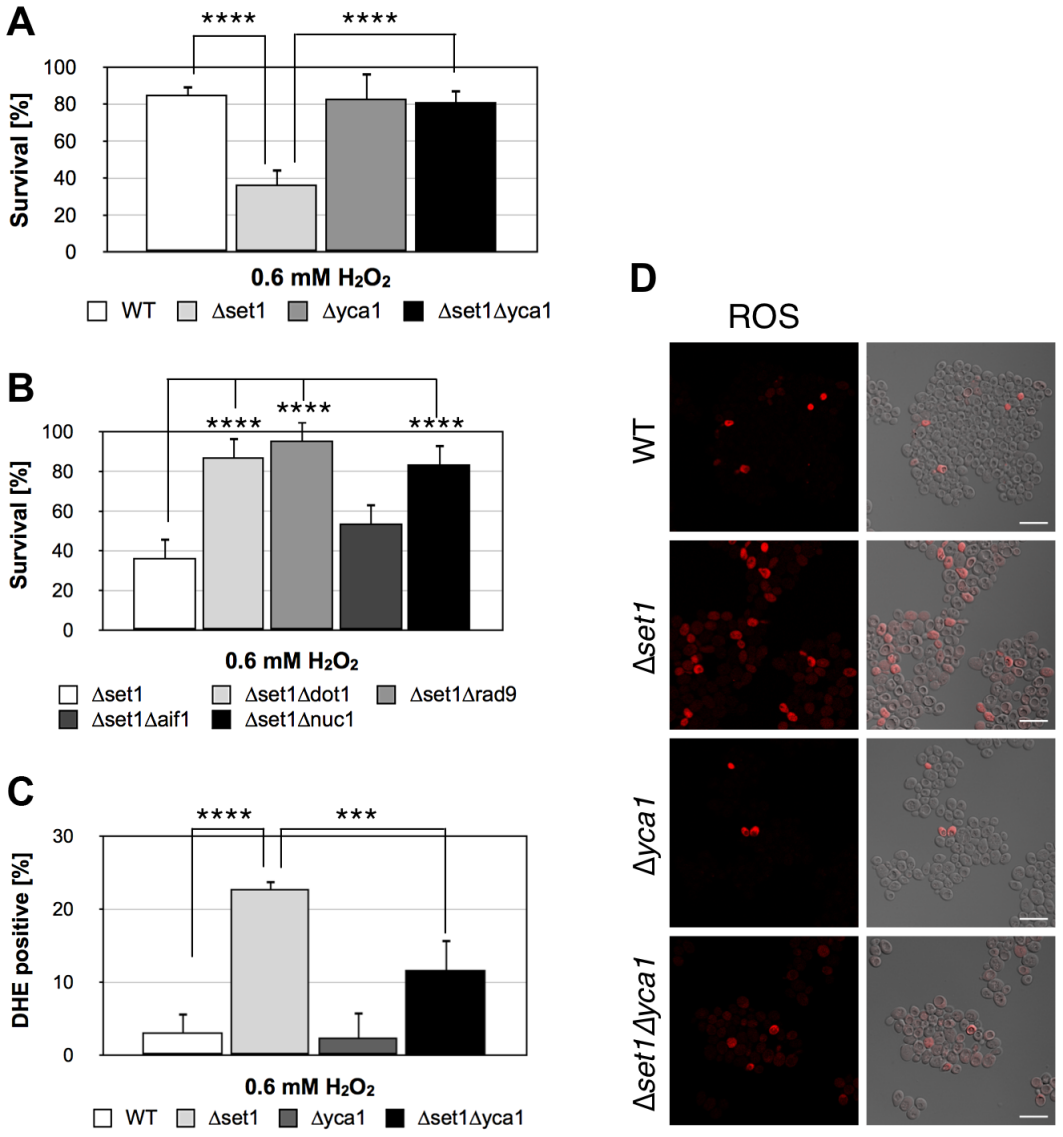
Disruption of H3K4 trimethylation sensitizes yeast to apoptotic stimuli. (**A**) Survival of wild-type (WT), *Δset1, Δyca1, and Δset1Δyca1* cells as well as (**B**) *Δset1, Δset1Δdot1, Δset1Δrad9, Δset1Δaif11, and Δset1Δnuc1* cells after treatment with 0.6 mM H_2_O_2_ for 8 hours. Cells were grown and treated in YPD medium. Data represent mean ± SD (*n* = 3, ****P<0.0001, **P<0.01). (**C**) DHE-positive WT, *Δset1, Δyca1, and Δset1Δyca1*cells were quantified after H_2_O_2_ treatment by manual counting of (**D**) fluorescence images. In each experiment, 2000–6000 cells were evaluated. Data represent mean ± SD, ****P<0.0001, ***P<0.001. Scale bars, 10 µm.

In order to rule out that the limited survival of *Δset1* cells during chronological aging is due to acidification of the medium and/or metabolic effects [53] and to demonstrate the importance of H3K4 trimethlyation in apoptosis regulation in more general, we induced apoptosis in *Δset1* cells using low concentration of hydrogen peroxide (H_2_O_2_). Whereas about 80% of wild-type and *Δyca1* cells were recovered after treatment of cells with 0.6 mM H_2_O_2_ for 8 hours, less than 40% of *set1* cells survived (Figure 8A). The reduced survival of *set1* cells coincided with increased ROS production (Figure 8C and D, Table 1). As during chronological aging, disruption of Y*CA1* in the *Δset1* cells conferred resistance to apoptosis induced by H_2_O_2_ (Figure 8A, C and D, Table 1). Similarly, double disruptants of *SET1* and *DOT1*, *RAD9*, and *NUC1*, respectively, were less sensitive to H_2_O_2_, whereas deletion of *AIF1* could not rescue the lethal effect of H_2_O_2_ on *Δset1* cells (Figure 8B). Our data therefore suggest that loss of H3K4 methylation leads to increased apoptosis and sensitizes cells to apoptotic stimuli.

## Discussion

Covalent histone modifications alter chromatin structure and DNA accessibility, which is playing important roles in a wide range of DNA-based processes, such as transcription regulation and DNA repair, but also cell division and apoptosis. In this context, particular changes in phosphorylation and acetylation of histones have been associated with the apoptotic process [54]. Moreover, H2B ubiquitination is important for nucleosome stability and its loss sensitizes yeast to nucleases [55] and to metacaspase-dependent cell death [39]. Histone H2B ubiquitination is a prerequisite for histone H3 K4 and K79 methylation, and this highly conserved trans-histone crosstalk has gathered much attention in recent years, since H3K4 and H3K79 methylation have been implicated in a variety of nuclear processes, such as transcription regulation, DNA replication, recombination and repair [22], [23], [56]. To further explore our prior study, we asked here whether a lack of H3K4 and/or H3K79 methylation affects apoptotic death of yeast cells and uncover the loss of H3K4 methylation as a novel apoptotic trigger.

### Loss of H3K4 methylation reduces the lifespan of chronologically aged yeast cells

**Figure 9:**
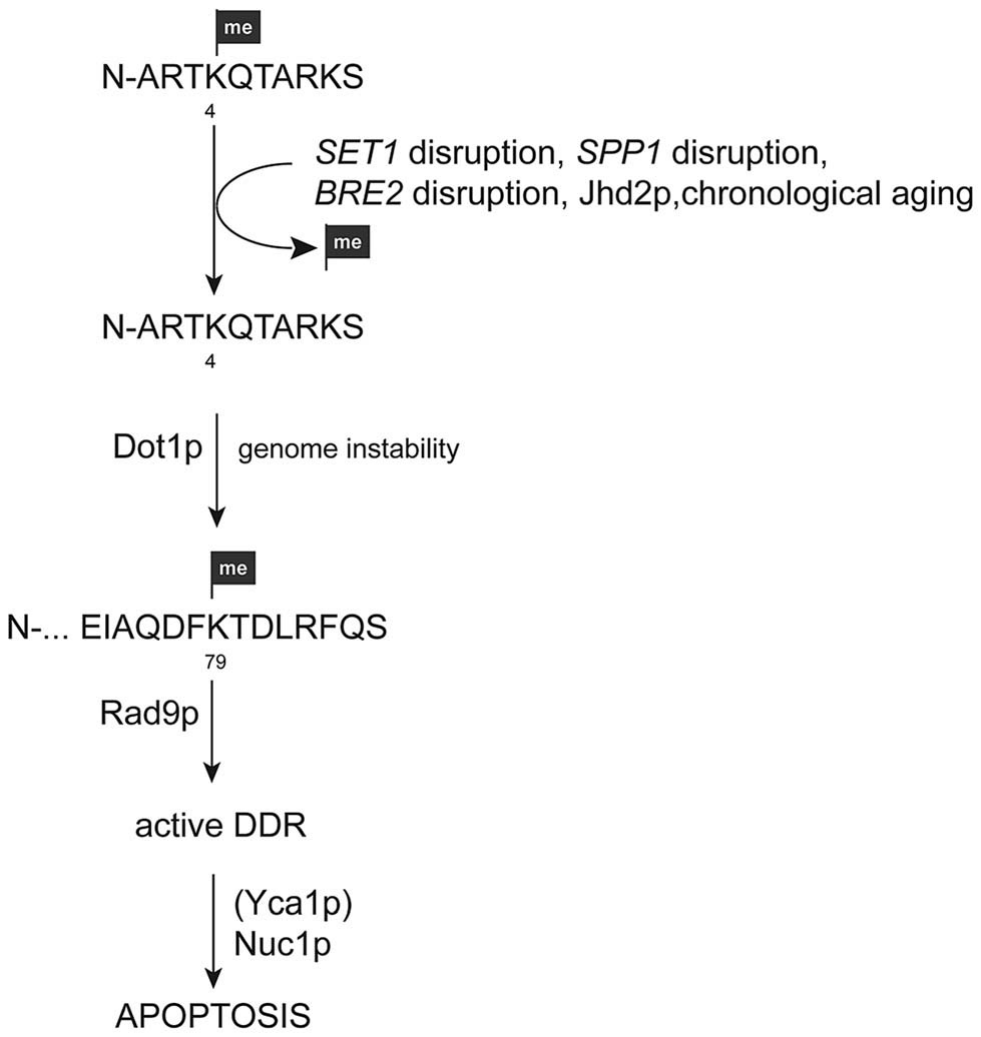
Model for apoptosis activation in yeast cells upon loss of H3K4 methylation. Loss of H3K4 methylation due to *SET1*, *SPP1*, or *BRE2* disruption, chronological aging, or the presence of the demethylase Jhd2p leads to genome instability. In the presence of the H3K79 methyl mark this leads to the recruitment of the DNA damage adaptor protein Rad9p and the activation of DNA damage response (DDR) machinery. Insufficient repair will in consequence cause an apoptotic response, which is in part Yca1p-dependent and conducted by executioners, such as Nuc1p.

Methylation of histone H3 at lysine 4 and 79 is accomplished by the evolutionary conserved methyltransferases Set1p and Dot1p, respectively [57], [58]. Methylation of both lysine residues appears to be associated with yeast cell death as loss of H3K4 methylation due to *SET1*, *SPP1* or *BRE2* deletion accelerated apoptosis (Figure 1 and 7), while disruption of *DOT1* and loss of H3K79 methylation delayed death of aged yeast cells (Figure 2). Similar results were obtained with the respective histone point mutants (Figure 7 E–H), indicating that H3K4 methylation is an anti-apoptotic mark, whereas H3K79 methylation is a pro-apoptotic mark. The loss of *SET1* and H3K4 methylation becomes apoptotic only in the presence of Dot1p and H3K79 methylation and can be suppressed by co-disruption of *DOT1* (Figure 3), which is likely linked to the DNA damage checkpoint. H3K79 methylation is important for the recruitment of the checkpoint adaptor protein Rad9p, the *S. cerevisiae* homolog of 53BP1, at damaged sites and for subsequent Rad53p phosphorylation to allow accurate DNA repair [32]. *SET1* disruptants rapidly accumulate mutations (Figure 4D) indicative of genome instability (see also [56]) and accelerated DNA damage, which activates the apoptotic machinery after checkpoint activation and failed repair. In the absence of Dot1p and H3K79 methylation, Rad9p recruitment to damaged sites and Rad53p phosphorylation is impaired, the DNA damage checkpoint is not or insufficiently activated, and consequently apoptosis is not activated irrespective to the state of DNA damage. In keeping with this, the co-disruption of *RAD9* in *Δset1* cells rescued survival (Figure 4A and B), despite the fact that the *Δset1Δrad9* cells exhibited a high mutation frequency (Figure 4D). Besides co-disruption of *DOT1* and *RAD9*, respectively, also co-deletion of Y*CA1* consequently suppressed the lethality of *Δ* *set1 cells*, at least in part (Figure 1). Yca1p is known as yeast metacaspase and numerous cell death scenarios depend on it [2], [8]. Yca1p likely acts downstream of the DNA damage checkpoint and insufficient DNA repair leads to its activation (Figure 9).

Another executioner of apoptosis in yeast is the endonuclease Nuc1p. Nuc1p can be activated independent of Yca1p and both proteins/pathways converge at the mitochondria [47]. Nuc1p translocates from mitochondria into the nucleus upon activation to degrade chromatin. Changes in chromatin structure due to loss of H3K4 methylation in the absence of Set1p may render yeast cells more sensitive to nuclease activity and consequently *NUC1* disruption in the *Δset1* background improved cell viability (Figure 5), in contrast to disruption of *AIF1* (Figure S1), which also exhibits nuclease activity [7]. Together our data presented here therefore suggest that loss of Set1p-mediated H3K4 methylation causes changes in chromatin structure and genomic instability, which activates the Rad9p-mediated DNA damage checkpoint in dependency on H3K79 methylation. Accumulation of DNA damage and insufficient repair in turn leads to an apoptotic response of the cells, which is executed by Yca1p (in part) and Nuc1p (Figure 9). Deletion of Dot1p and the loss of H3K79 methylation blocks activation of the DNA damage checkpoint and subsequent apoptosis.

### Loss of H3K4 methylation triggers cell death during chronological aging

Apoptosis in yeast can be triggered exogenously and endogenously. Known endogenous triggers are, for example, defects in DNA damage response and replication, chromatin condensation, mRNA stability, or N-glycosylation [46], [59]–[62]. Chronological aging of yeast cells is the best-studied physiological scenario associated with apoptosis in *S. cerevisiae* and the lifespan of aged yeast cells can be prolonged or shortened in many ways [4], [63], [64]. Glucose and nutrients have a strong impact on the CLS of yeast [63], [65], whereas endogenous triggers, however, have remained largely unknown. Our data presented here suggest loss of H3K4 methylation as one such endogenous trigger. Wild-type yeast cells lost H3K4 tri- and dimethylation (Figure 6A) after 6 days of culturing, which coincided with a significant increase in cell death (Figure 1A). In contrast to that, H3K79 methylation is not altering during chronological aging. Preventing demethylation by either deleting *DOT1* (Figure 6B) or deleting the trimethyl demethylase Jhd2p (Figure 6C and D) delayed against age-induced cell death, indicating that loss of H3K4 methylation is sufficient to drive yeast cells into apoptosis. Particularly, the loss of H3K4 trimethylation seems to promote apoptosis as *Δjhd2* cells have low to no H3K4me2 levels (Figure 6A), similar to *Δset1* cells. Loss of H3K4me3 is not only triggering apoptosis, but also sensitizes yeast cells to apoptotic stimuli such as exposure to H_2_O_2_ (Figure 8A–D), further underlying the importance of this histone modification in apoptosis regulation. H2B ubiquitination is required for H3K4 and H3K79 methylation and it remains to be seen if changes in H2B ubiquitination are the cause for the suppression of H3K4 demethylation upon disruption of *DOT1* or if the recruitment of Jhd2p to H3K4 methylation is hindered in the absence of H3K79 methylation. This will be subject of future investigation.

Given the strong evolutionary conservation of H3K4 and H3K79 methylation by the Set1/COMPASS complex and Dot1, respectively, our findings pinpoint to a contribution of a deregulated apoptotic response to the pathology of acute myeloid leukemia (AML). AML is associated with chromosomal translocations involving the *MLL* gene, the human homolog of Set1p. MLL-associated leukemia are aggressive, characterized by a frustrating therapy outcome, and are DOT1L-dependent [66]. It will be interesting to see how much our findings described here apply to human cells, especially to hematopoietic cells.

## Materials and Methods

### Plasmids, yeast strains, and culture conditions

BY4742 (MATα; his3*Δ*1; leu2*Δ*0; lys2*Δ*0; ura3*Δ*0) and its derivatives *Δdot1*, *Δyca1*, *Δrad9*, *Δnuc1*, *Δjhd2*, *Δ aif1*, *Δspp1*, and *Δbre2* were obtained from Euroscarf. *Δset1* was derived from BY4742, *Δset1Δdot1* and *Δset1Δdot1Δyca1* were derived from *Δdot1, Δset1Δyca1* and *Δdot1Δyca1* were derived from *Δyca1*, *Δset1Δrad9* and *Δset1Δrad9Δdot1* were derived from *Δrad9*, *Δset1Δnuc1* were derived from *Δnuc1*, and *Δset1Δaif1* were derived from *Δaif1* strains. All derivate strains were constructed according to [67] PCR-based gene deletion. All strains are listed in Table 2.

**Table 2:** Yeast strains used in this study.

Yeast strain	Genotype	Source
BY4742	*MATα, his3Δ1, leu2Δ0, lys2Δ0, ura3Δ0*	Euroscarf
BFY315	*MATα, his3Δ1, leu2Δ0, lys2Δ0, ura3Δ0, rad9Δ::kanMX4*	Euroscarf
BFY317	*MATα, his3Δ1, leu2Δ0, lys2Δ0, ura3Δ0, yca1Δ::kanMX4*	Euroscarf
BFY426	*MATα, his3Δ1, leu2Δ0, lys2Δ0, ura3Δ0, aifΔ::kanMX4*	Euroscarf
BFY427	*MATα, his3Δ1, leu2Δ0, lys2Δ0, ura3Δ0, nuc1Δ::kanMX4*	Euroscarf
BFY503	*MATα, his3Δ1, leu2Δ0, lys2Δ0, ura3Δ0, dot1Δ::kanMX4*	Euroscarf
BFY622	*MATα, his3Δ1, leu2Δ0, lys2Δ0, ura3Δ0, jhd2Δ::kanMX4*	Euroscarf
BFY632	*MATα, his3Δ1, leu2Δ0, lys2Δ0, ura3Δ0, spp1Δ::kanMX4*	Euroscarf
BFY633	*MATα, his3Δ1, leu2Δ0, lys2Δ0, ura3Δ0, bre2Δ::kanMX4*	Euroscarf
BFY405	*MATα, his3Δ1, leu2Δ0, lys2Δ0, ura3Δ0, set1Δ::loxP-LEU2-loxP*	This study
BFY414	*MATα, his3Δ1, leu2Δ0, lys2Δ0, ura3Δ0, dot1Δ::kanMX4, set1Δ::loxP-LEU2-loxP*	This study
BFY440	*MATα, his3Δ1, leu2Δ0, lys2Δ0, ura3Δ0, yca1Δ::kanMX4, set1Δ::loxP-LEU2-loxP*	This study
BFY478	*MATα, his3Δ1, leu2Δ0, lys2Δ0, ura3Δ0, dot1Δ::kanMX4, set1Δ::loxP, yca1ΔloxP-LEU2-loxP*	This study
BFY480	*MATα, his3Δ1, leu2Δ0, lys2Δ0, ura3Δ0, dot1Δ::kanMX4, yca1ΔloxP-LEU2-loxP*	This study
BFY563	*MATα, his3Δ1, leu2Δ0, lys2Δ0, ura3Δ0, rad9Δ::kanMX4, set1Δ::loxP-LEU2-loxP*	This study
BFY576	*MATα, his3Δ1, leu2Δ0, lys2Δ0, ura3Δ0, dot1Δ::kanMX4, set1Δ::loxP, rad9ΔloxP-LEU2-loxP*	This study
BFY619	*MATα, his3Δ1, leu2Δ0, lys2Δ0, ura3Δ0, nuc1Δ::kanMX4, set1Δ::loxP-LEU2-loxP*	This study
BFY626	*MATα, his3Δ1, leu2Δ0, lys2Δ0, ura3Δ0, aif11Δ::kanMX4, set1Δ::loxP-LEU2-loxP*	This study
BFY649	*MATa, his3Δ200, leu2Δ0, lys2Δ0, trp1Δ63, ura3Δ0, met15Δ0, can1::MFA1pr-HIS3, hht1-hhf1::NatMX4, hht2-hhf2::URA3 [pJP11 CEN-LYS2, HHT1-HHF1]*	Open Biosystems
BFY650	*MATa, his3Δ200, leu2Δ0, lys2Δ0, trp1Δ63, ura3Δ0, met15Δ0, can1::MFA1pr-HIS3, hht1-hhf1::NatMX4, hht2-hhf2::URA3 [pCEN-LYS2 hht1K4A-HHF1]*	Open Biosystems
BFY651	*MATa, his3Δ200, leu2Δ0, lys2Δ0, trp1Δ63, ura3Δ0, met15Δ0, can1::MFA1pr-HIS3, hht1-hhf1::NatMX4, hht2-hhf2::URA3 [pCEN-LYS2 hht1K79A-HHF1]*	Open Biosystems

Survival plating was conducted on YPAD (1% yeast extract, 2% peptone, and 2% glucose, 40 mg/ml adenine) media supplemented with 2% agar. For experiments testing the chronological lifespan, strains were grown in synthetic complete medium (SC) with 2% glucose [68].

Transformation of yeast cells was performed by the lithium acetate procedure, as described by [69].

### Chronological aging and test for apoptotic markers

Chronological aging experiments, hydrogen peroxide treatment, and apoptotic tests using DHE-staining and TUNEL-staining were performed as described previously [39]. All chronological aging experiments reported were conducted at least three times, with three replicates for each strain. Integrals of the life span curves were calculated by summing the trapezoids created by the viability time points as described previously [40]. For the calculation of integrals 100% survival was set as 1. P values were assigned by calculating the variance of integrals between biological replicates and comparing this to the integrals for wild-type cells using a T-test. Cells were viewed using a Leica TCS SP5 and a Zeiss LSM 710 confocal laser scanning microscope. Images were recorded using the microscope system software and processed using Image J and Adobe Photoshop. For quantification of DHE-staining using flow cytometry (FACS-Aria, BD), in each sample 10.000 cells were evaluated and processed using BD FACSDiva software. For Annexin V staining the Annexin-V-Fluos staining kit (Roche, Basel, Switzerland) was used, following the instructions of the manufacturer.

### Measurements of mutation frequency

Spontaneous mutation frequency was determined based on the appearance of mutants able to form colonies on agar plates containing 60 mg l^−1^ L-canavanine sulfate according to [46]. Mutation rates were calculated per 10^6^ living (colony forming on YPD) cells.

### Immunoblotting

Cell extract were prepared by acid extraction using 10% trichloroacetic acid (TCA) according to [70]. In brief, 1.5 ml culture were pelleted by centrifugation at 4°C and frozen at −20°C. 150 µl TCA buffer (10 mM Tris, pH 8.0, 10% TCA, 25 mM ammonium-acetate, 1 mM EDTA) were added to the frozen pellet on ice. When thawed, half the volume glass beads were added and samples were vortex 5×1 min with 3 min intervals on ice in between. The cell lysates were then transferred into a fresh, pre-cooled microfuge tube on ice and centrifuged for 10 min at 16.000 g at 4°C. The supernatant was discarded, the pellet resuspended in 100 µl resuspension solution (0.1 M Tris, pH 11.0, 3%SDS), and boiled for 5 min. After cooling to room temperature, the samples were spun for 30 sec at 16.000 g to pellet the cell debris and 80 µl were transferred into a fresh microfuge. Protein concentrations were determined using the Bio-Rad DC protein assay (Bio-Rad, Munich, Germany) and 30 µg of proteins per well were loaded onto a 15% gel. After SDS-PAGE, proteins were transferred to a PVDF membrane and membranes were probed with the following rabbit polyclonal antibodies: anti-histone H3K4me3 (1∶1000 dilution; 39915, Active Motif), anti-histone H3K4me2 (1∶1000; 39141, Active Motif), anti-histone H3K79me3 (1∶1000; ab2621, Abcam), anti-histone H3 (1∶500; 9715; Cell Signaling), the mouse monoclonal anti-PGK antibody (1∶10.000; Invitrogen) and the respective alkaline-phosphatase conjugated secondary antibodies (1∶20.000; Sigma-Aldrich). Membranes were developed using the Western Lightning CDP-Star Chemiluminescence Reagent (Tropix) and X-ray films. The films were scanned and processed using Adobe Photoshop. Densitometric quantification was performed from three independent experiments using Image J.

Human lymphocyte cell lines were obtained from Coriell Institute (Coriell Institute, Camden, NJ, USA). Cell were grown in suspension in RPMI 1640 medium supplemented with 15% FBS and 2 mM L-glutamine. Cells were cultured at 37°C/5% CO_2_. Cells were harvested by centrifugation at 600× g for 5 min. The pelleted cells were washed in PBS, resuspended in lysis buffer containing 50 mM Tris-HCl, pH 7.8, 150 mM NaCl, 1% Nonidet P-40 and protease inhibitor cocktail tablets (Roche, Basel, Switzerland). 30 µg of proteins per well were loaded onto a 15% gel and SDS-PAGE and Western blotting was carried out as described above.

## Supporting Information

Figure S1 Yeast cells lacking Set1p do not benefit from *AIF1* disruption. (**A**) Survival of WT, *Δset1*, *Δaif1* and *Δset1Δaif1* cells was determined by clonogenicity during chronological aging over 14 days (**B**). Integrals under the life span curves were determined. Data represent mean ± SD (n = 3, ***P<0.001, ****P<0.0001). (**C**) DHE-positive WT, *Δset1*, *Δaif1* and *Δset1Δaif1* cells were quantified after 6 days in culture by fluorescence microscopy. In each experiment, 2000–4000 cells were evaluated. Data represent mean ± SD, *P<0.05, ***P<0.001, ****P<0.0001. (**D**) ROS accumulation in WT, *Δset1*, *Δaif1* and *Δset1Δaif1* cells after six days in culture was determined by DHE staining and visualized by fluorescence microscopy. Scale bars, 10 µm.
(PDF)

Figure S2 Disruption of *DOT1* and *JHD2* prolong the chronological life span of yeast cell moderately. (**A**) Survival of WT and *Δdot1* cells was determined by clonogenicity during chronological aging over 10 days and normalized to 100% survival at day 2 (**B**). Integrals under the life span curves were determined: integral 5.1 for WT cells versus 5.6 for *dot1* depleted cells. Data represent mean ± SD (n = 3, *P<0.05). (**C**) Survival of WT and *Δjhd2* cells was determined by clonogenicity during chronological aging over 13 days and normalized to 100% survival at day 2 (**D**). Integrals under the life span curves were determined: integral 6.1 for WT cells versus 7.1 for *jhd2* depleted cells. Data represent mean ± SD (n = 3, *P<0.05).
(PDF)
