# Supplementary figures and images for: Loss of Histone H3 Methylation at Lysine 4 Triggers Apoptosis in Saccharomyces cerevisiae
Source: PLoS Genet. 2014 Jan 30;10(1):e1004095. doi: 10.1371/journal.pgen.1004095 (PMC3907299; doi:10.1371/journal.pgen.1004095)

Figure S1

**A**

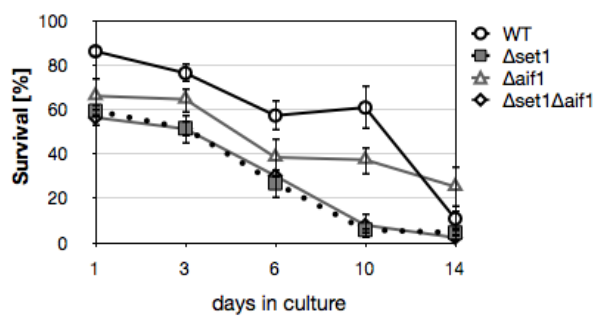

**B**

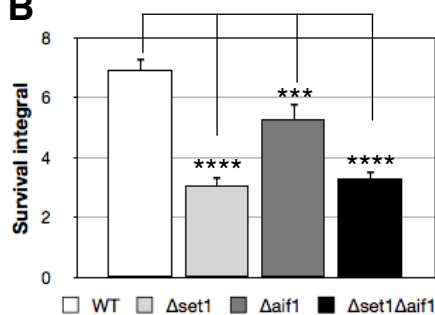

**C**

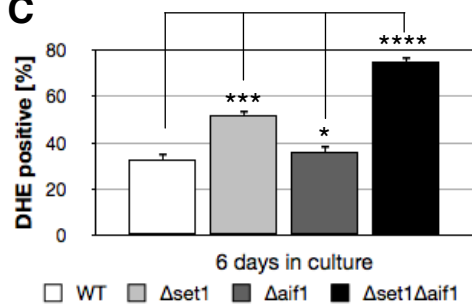

**D**

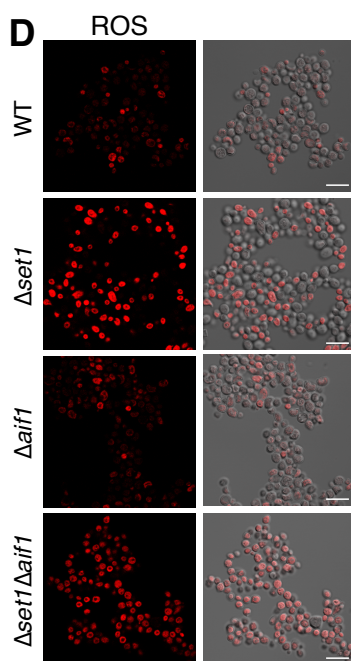

Supplement: Figure S1 — Yeast cells lacking Set1p do not benefit from AIF1 disruption. (A) Survival of WT, Δset1, Δaif1 and Δset1Δaif1 cells was determined by clonogenicity during chronological aging over 14 days (B). Integrals under the life span curves were determined. Data represent mean ± SD (n = 3, ***P<0.001, ****P<0.0001). (C) DHE-positive WT, Δset1, Δaif1 and Δset1Δaif1 cells were quantified after 6 days in culture by fluorescence microscopy. In each experiment, 2000–4000 cells were evaluated. Data represent mean ± SD, *P<0.05, ***P<0.001, ****P<0.0001. (D) ROS accumulation in WT, Δset1, Δaif1 and Δset1Δaif1 cells after six days in culture was determined by DHE staining and visualized by fluorescence microscopy. Scale bars, 10 µm. (PDF) [file pgen.1004095.s001.pdf]

Figure S2

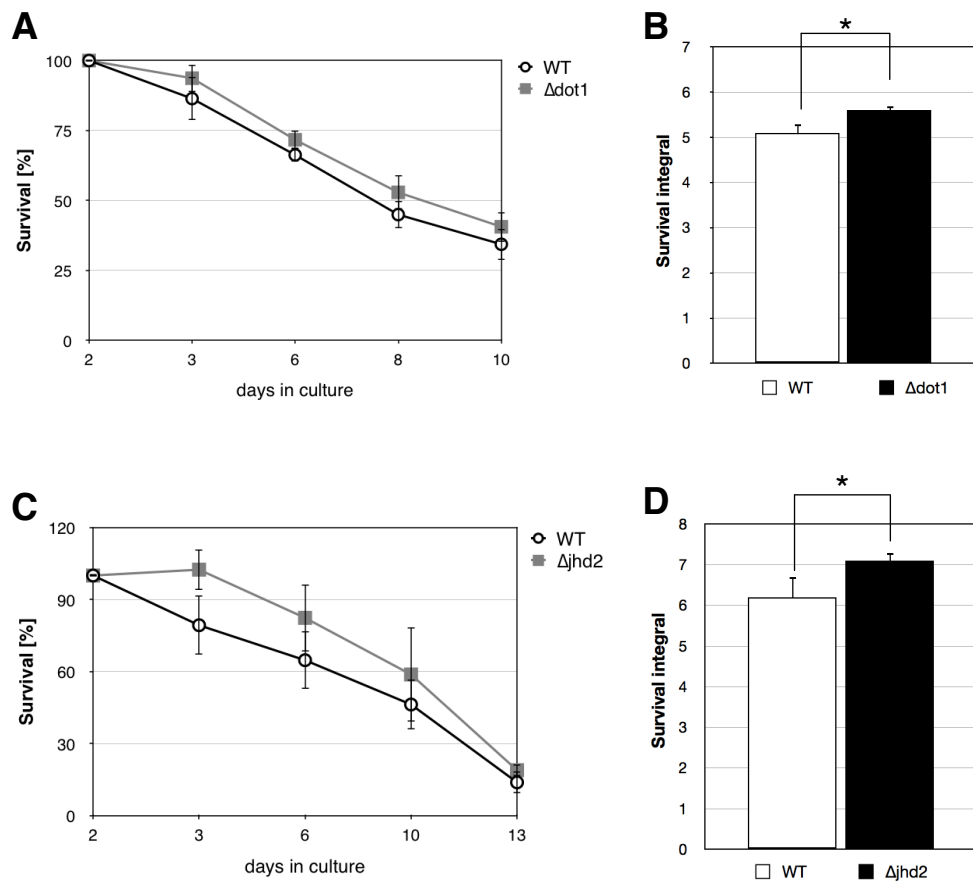

Supplement: Figure S2 — Disruption of DOT1 and JHD2 prolong the chronological life span of yeast cell moderately. (A) Survival of WT and Δdot1 cells was determined by clonogenicity during chronological aging over 10 days and normalized to 100% survival at day 2 (B). Integrals under the life span curves were determined: integral 5.1 for WT cells versus 5.6 for dot1 depleted cells. Data represent mean ± SD (n = 3, *P<0.05). (C) Survival of WT and Δjhd2 cells was determined by clonogenicity during chronological aging over 13 days and normalized to 100% survival at day 2 (D). Integrals under the life span curves were determined: integral 6.1 for WT cells versus 7.1 for jhd2 depleted cells. Data represent mean ± SD (n = 3, *P<0.05). (PDF) [file pgen.1004095.s002.pdf]
